# The role of cellular contact and TGF-beta signaling in the activation of the epithelial mesenchymal transition (EMT)

**DOI:** 10.1080/19336918.2018.1526597

**Published:** 2018-10-08

**Authors:** Kelsey Gasior, Nikki J. Wagner, Jhon Cores, Rose Caspar, Alyson Wilson, Sudin Bhattacharya, Marlene L. Hauck

**Affiliations:** a Biomathematics Program, North Carolina State University, Raleigh, NC, USA; b College of Veterinary Medicine, North Carolina State University, Raleigh, NC, USA; c Joint Department of Biomedical Engineering, University of North Carolina and North Carolina State University, Chapel Hill, NC, USA; d Department of Statistics, North Carolina State University, Raleigh, NC, USA; e Department of Biomedical Engineering, Michigan State University, East Lansing, MI, USA; f Department of Pharmacology & Toxicology, Michigan State University, East Lansing, MI, USA; g Center for Research on Ingredient Safety, Michigan State University, East Lansing, MI, USA; h Institute for Quantitative Health Science and Engineering, Michigan State University, East Lansing, MI, USA; i Institute for Integrative Toxicology, Michigan State University, East Lansing, MI, USA

**Keywords:** EMT, TGF-β, cellular adhesion, epithelial, mesenchymal, breast carcinoma, colon carcinoma

## Abstract

The epithelial mesenchymal transition (EMT) is one step in the process through which carcinoma cells metastasize by gaining the cellular mobility associated with mesenchymal cells. This work examines the dual influence of the TGF-β pathway and intercellular contact on the activation of EMT in colon (SW480) and breast (MCF7) carcinoma cells. While the SW480 population revealed an intermediate state between the epithelial and mesenchymal states, the MC7 cells exhibited highly adhesive behavior. However, for both cell lines, an exogenous TGF-β signal and a reduction in cellular confluence can push a subgroup of the population towards the mesenchymal phenotype. Together, these results highlight that, while EMT is induced by the synergy of multiple signals, this activation varies across cell types.

## Introduction

The most common type of cancer found in humans are carcinomas, tumors arising from epithelial cells [1,2]. In both normal tissue and carcinomas, epithelial cells utilize a transmembrane protein complex involving E-cadherin and members of the catenin family to establish strong cellular bonds with their neighboring cells [3,4]. These strong cell-cell bonds inhibit individual cell movement and give epithelial cells their characteristic adhesive properties [5].

Cells in a carcinoma can undergo the epithelial to mesenchymal transition (EMT) [6], wherein an epithelial cell loses its adhesion with its surroundings and adopts the invasive and migratory behavior that is characteristic of mesenchymal cells [7,8]. While this process is beneficial during embryogenesis and wound healing, tumor cells acquiring these characteristics can lead to lethal consequences [6,9–11]. Characterized by their spindle-like phenotype [5], these newly formed mesenchymal cells can invade the microenvironment surrounding the tumor, enter the blood stream, and travel to a distant site in the body. Once at this distant site, these mesenchymal cells can undergo the reverse process, the mesenchymal epithelial transition (MET), proliferate, and form a metastasis [6].

Many pathways have been implicated in EMT. We have recently modeled the role of the Wnt signaling pathway in EMT activation and the underlying biological switch [12]. Another of the main pathways activating EMT *in vivo* is the transforming growth factor β (TGF-β) pathway [13,14]. In the tumor environment, the binding of TGF-β to TGF-β receptors (TGFβR) on the carcinoma cell initiates an intracellular protein cascade [15,16]. TGF-β binding to TGFβR leads to the phosphorylation of the R-Smads: Smad2 and Smad3 [15]. These newly phosphorylated R-Smads then associate with the Co-Smad, Smad4, and the complex translocates to the nucleus [17]. Once in the nucleus, the Smad complex upregulates genes of the Snail family, such as Slug [17,18]. Slug then binds with E-boxes in the E-cadherin promoter and suppresses the transcription of E-cadherin [18,19]. The suppression of E-cadherin decreases the amount of adhesion complex molecules available to form cell-to-cell bonds. Additionally, Slug contributes to the relocation of E-cadherin from the membrane to the cytosol in epithelial cells [18], further depleting the presence of the cellular adhesion complex and thus pushing the cell towards the mesenchymal phenotype.

Previous work has shown that a loss of cellular junctions, and subsequent cellular contact, is associated with increased invasiveness in carcinoma cells. This loss of cellular adhesion results from a decrease or loss of function in E-cadherin and, it has been shown that restoring E-cadherin function can cause a cell to revert back to its non-invasive behavior [1,20,21]. Although the activation of the TGF-β pathway suppresses E-cadherin and ultimately reduces the extent of cell-cell contact, it is our hypothesis that existing intercellular contact prevents or delays the activation of EMT. Specifically, we believe existing cell-cell contact encourages the epithelial phenotype and related behavior in cells by promoting functional and active intercellular junctions, thereby making it more difficult for the cell to undergo EMT via TGF-β pathway activation. In this study, we test this competition between extracellular pro-epithelial (cell-cell contact) and pro-mesenchymal (TGF-β) cues in both SW480 colon carcinoma cells [22] and MCF7 breast carcinoma cells [20,23].

## Materials and methods

### Cell culture

SW480 (human colorectal adenocarcinoma) and MCF7 (human breast adenocarcinoma) cells were cultured and maintained in Dulbecco’s Modification of Eagle’s Medium (DMEM: Mediatech) supplemented with penicillin-streptomycin, L-glutamine (Mediatech), Plasmocin prophylactic (InvivoGen), and 10% heat inactivated fetal bovine serum (FBS, Mediatech).

SW480 Confluency: In order to obtain 30% confluency, 2.71 × 10^5^ – 1.35 × 10^6^ were seeded into a 143 cm^2^ tissue culture treated dish and grown to an estimated 20–40% confluency. For 60% confluency, 5.41 × 10^5^ – 2.71 × 10^6^ cells per dish were grown to an estimated 50–70% confluency. 5.41 × 10^6^ – 2.71 × 10^7^ cells per dish were grown to 90–100% confluency.

MCF7 Confluency: Cells were grown in 143 cm^2^ tissue culture treated dishes: 2.38 × 10^5^ – 7.92 × 10^5^ cells were seeded to each dish and grown to an estimated 30% confluency. 7.92 × 10^5^ – 1.58 × 10^6^ cells were seeded to each dish and grown to an estimated 50–70% confluency, and 2.38 × 10^6^ – 1.19 × 10^7^ cells were grown to 90–100% confluency.

For microscopy experiments, cells were grown in four-well chamber slides (Millipore Sigma, PEZGS0416). For all SW480 treatment groups, 2 × 10^3^ cells were seeded per well. For MCF7 treatment groups at low confluence, 5 × 10^3^ cells were seeded per well; for those at high confluence treatment groups, 5 × 10^3^–5 × 10^4^ cells were seeded per well.

For TGF-β stimulation, cells were incubated in media with 1% FBS for 24 hours before being given fresh media containing 1% FBS and either 3 ng/mL or 9.33 ng/mL of TGF-β (R&D Systems, 240-B-010) and incubated for 48 additional hours.

### Protein extractions

Cells were collected from tissue culture dishes using a cell scraper. For 100% confluent treatment groups, cells were collected from the entire dish. For dishes that were part of the 30% and 60% treatment groups, cells were only taken from the center of the plate where confluence was truly 20–40% or 50–70% respectively. Cells were placed into single cell suspension using a 22-gauge needle, counted, and 2.5 – 5.5 × 10^7^ cells were collected and pelleted. Differential detergent fractionation (DDF) protein extraction adapted from McCarthy et al. [24] was performed. For each extraction, the Sequential Detergent Extraction (SDE) Buffer 1 was prepared fresh as described in McCarthy et al. [24] with a final concentration of 25% Base Buffer 1. One mL of SDE Buffer 1 was added to the cell pellet. The cells were resuspended in the buffer using gentle pipetting and then incubated on ice for 30 min with gentle mixing using a rocker. Samples were centrifuged for 5 min at 550×g and 4°C. The supernatant was removed and then centrifuged for 10 min at 10600×g and 4°C. The supernatant was then collected and held on ice. These steps were repeated 9 times for a total of 10 extractions. All supernatants were combined and stored at −80°C.

### TGF-β ELISA

Cytosolic TGF-β1: TGF-β1 concentration was measured using the Quantikine ELISA Human TGF-β1 Kit from R&D Systems (SB100B) as described by the manufacturer.

### Quantitative polymerase chain reaction (qPCR)

RNA Extraction and cDNA Preparation: Cells were collected in the same manner as described in the Protein Extraction section and RNA extractions were carried out following the OMEGA bio-tek E.Z.N.A. Total RNA Kit I Animal Cell Protocol was followed as described by the manufacturer. For each sample, 1.5 × 10^6^ – 3 × 10^6^ cells were collected and RNA was eluted in 40 μL DEPC H_2_O. A concentration of 2 μg/20 μL of cDNA was then synthesized from the RNA using the SuperScript VILO cDNA Synthesis Kit Protocol for First-Strand cDNA Synthesis (Invitrogen). RNA quality was checked using a NanoDrop and Agilent Bioanalyzer.

qPCR: Primer sequences for both reference genes (RPS17 and PUM1) [25–28] and both genes of interest (E-cadherin and Slug) [29] are listed in Table 1. Specificity of primers was confirmed using the NCBI Basic Local Alignment Search Tool (BLAST). The optimal primer concentration for this assay was 200nM for each primer and primer efficiencies were determined using a standard curve of diluted DNA. Calculated primer efficiencies ranged from 103% to 112%.

**Table 1.** qPCR primers.

Gene	Forward Sequence	Reverse Sequence	Refs
RPS17	AAGCGCGTGT-GCGAGGAGATCG	TCGCTTCATCAGATGC-GTGACATAACCTG	[26]
PUM1	TGAGGTGTGCA-CCATGAAC	CAGAATGTGCTT-GCCATAGG	[24]
E-CADHERIN	CCCGGGACAA-CGTTTATTAC	GCTGGCTCAAG-TCAAAGTCC	[27]
SLUG	TGGTTGCTTCAA-GGACACAT	GTTGCAGTGAG-GGCAAGAA	[27]

qPCR reactions were run in triplicate using 12.5 ng of cDNA and Power SYBR Green PRC Master Mix (Thermo Fisher Scientific, 4367659) on the Step One RT PCR System (Applied Biosystems). Reactions were heated to 95.0°C for 10 minutes before 40 cycles of 95.0°C for 15 seconds and 60.0°C for 1 minute. Following cycling, a melt curve was generated. Cycle threshold (Ct) values with a standard deviation >0.5 were repeated and relative expression for both E-cadherin and Slug were calculated using their Ct values for the target and the reference genes.

### Flow cytometry

Cells were collected in the same manner as described in the Protein Extraction section. For each sample, 4 × 10^5^–1 × 10^6^ cells were stained. First, live cells were identified by adding 1 μL of eBioscience Fixable Viability Dye was added per 1 × 10^6^ cells for 30 minutes at 4°C in the dark. Cells were then centrifuged at 500xg at room temperature for 5 minutes, washed with FACS buffer (PBS containing 0.05% azide and 2% FBS), then stained with 5 μL of phycoerythrin (PE) mouse anti-human E-cadherin antibody (BD Biosciences, 562870) per 1 × 10^6^ cells for 30 minutes at 4°C. Cells were washed twice, then fixed and permeabilized using eBioscience IC Fixation Buffer and Permeabilization Buffers (Thermo Fischer Scientific, 88-8824-00) as described by the manufacturer. Cells were stained with 5 μL of Alexa 488 mouse anti-human vimentin antibody (BD Biosciences, 562338) per 1 × 10^6^ cells for 20 minutes at room temperature. Samples were washed once with 1X Permeabilization Buffer and once with FACS buffer. Finally, cells were resuspended in FACS buffer and analyzed on a BD LSRII flow cytometry (BD Biosciences). As a staining control, compensation beads (eBioscience, Thermo Fisher Scientific, 01-2222-41) were also stained with PE mouse anti-human E-cadherin stain and Alexa 488 mouse anti-human vimentin stain, and fixed following staining. Gating strategy is shown in Supplementary Figure 1.

### Immunocytochemistry

Cell slides were washed 1x with PBS and then fixed for 30 minutes at room temperature in 4% paraformaldehyde. Cells were then washed 3x with PBS and permeabilized and blocked with a solution of Dako Serum-free Protein Block and 0.1% saponin for 1 hour at room temperature. Mouse anti-human E-cadherin antibody (BD Biosciences, 562869) was diluted in the permeabilization solution, (1:50 for SW480 cells and 1:22.5 for the MCF7 cells), and slides were incubated overnight at 4°C. Following the incubation, the cells were washed in PBS, and incubated in diluted (1:220) Texas Red labeled goat anti-mouse IgG (H&L) (Abcam, ab6787) for 90 minutes. Following this incubation, cells were washed with PBS. Alexa 488 mouse anti-human vimentin (BD Biosciences, 562338) was diluted in the permeabilization solution to a ratio 1:50 and cells incubated overnight in the dark at 4°C. Following the incubation with the anti-vimentin antibody, the cells were washed and stained with DAPI for 10 minutes. Cells were given a final set of washes: two quick washes and then two 10 minute washes in PBS. Cells were mounted with Prolong Gold (Life Technologies, P36930) and a cover slide and set for at least 4 hours before imaging. For each treatment group (and each technical replicate), 15 sets of non-overlapping images were taken at 20x magnification: one image of the blue DAPI filter, one image of the Texas Red E-cadherin filter, and one image of the Alexa 488 vimentin filter, and one bright field image using an Olympus IX81 microscope and a Hamamatsu Orca-Flash4.0 V2 Digital CMOS camera 22cu.

Neighbor Number: Using ImageJ, the DAPI background was subtracted and the image was then converted to an 8.0bit image, see Supplemental Figure 2. Using the threshold and watershed features [30], nuclei were isolated (see Supplementary Figure 2A). Analyzing particles that ranged in size from 500-infinity and had a circularity of 0–1.0, the individual cellular nuclei were numbered and a drawing of the nuclei, as well as their corresponding numbers for each image was produced (see Supplementary Figure 2B). Additionally, using the analyze particles feature, ImageJ recorded the X and Y coordinates of the center of mass of each nucleus.

To gauge the distance between nuclei of neighboring cells, 5% of the cells from each of the 30% confluent + 0 ng/mL TGF-β treatment group were chosen for each cell line. Using the DAPI nuclei filtered image and the corresponding bright field image, the numeric neighbor of each cell and the distance between the center of mass of their two nuclei was recorded. For the SW480 cells, the neighboring nuclei were found to have an average distance of 26.59 μm and a standard deviation of 12.97 μm, while, in the MCF7 cells, the average distance between the nuclei of neighboring cells was 29.92 μm with a standard deviation of 12.53 μm. Using the MATLAB software, three threshold distances for each cell line were determined: the average cellular distance, the average cellular distance +1 standard deviation, the average cellular distance +2 standard deviations. Then, for each distance threshold, it was determined how many neighbors a given cell had.

Cellular Staining Determination: Once cellular neighbor number was established for each of the three threshold distances, cells were divided into three categories: low number of neighbors (0–2), medium number of neighbors (3–5), and high number of neighbors (6 or greater). Using the MATLAB software, 25 cells (or the maximum number of cells if less than 25) were randomly selected from each category for each treatment group. Using a control image for each stain, the brightness and contrast for each image were adjusted in ImageJ. At each distance threshold, the staining of the cells was determined for the chosen cells in each of the neighbor number categories.

### Statistics

TGF-β ELISA: A two-way ANOVA test was employed to analyze whether changes in confluence and exogenous TGF-β influenced the concentration of cytosolic TGF-β for each cell line. A value of α = 0.05 was used for analysis.

qPCR: Statistical analysis was carried out via the REST© software [31] which uses a Pair Wise Fixed Reallocation Randomisation Test© to determine whether or not there was a difference in expression between the control and treatment groups. A value of α = 0.05 was used for analysis.

Flow Cytometry: The flow cytometry data was converted into contingency tables with three levels of confluence, three concentrations of exogenous TGF-β, and four categories of staining. Chi Squared Tests of Homogeneity were carried out [32]. A value of α = 0.05 was used for analysis.

Immunocytochemistry: In order to analyze the impact of neighbor number and concentration of exogenous TGF-β on cellular staining, Chi Squared Tests of Homogeneity were used. A value of α = 0.05 was used for analysis.

## Results

### Cytosolic TGF-β level is dependent upon cellular confluence and exogenous TGF-β

**Figure 1.**
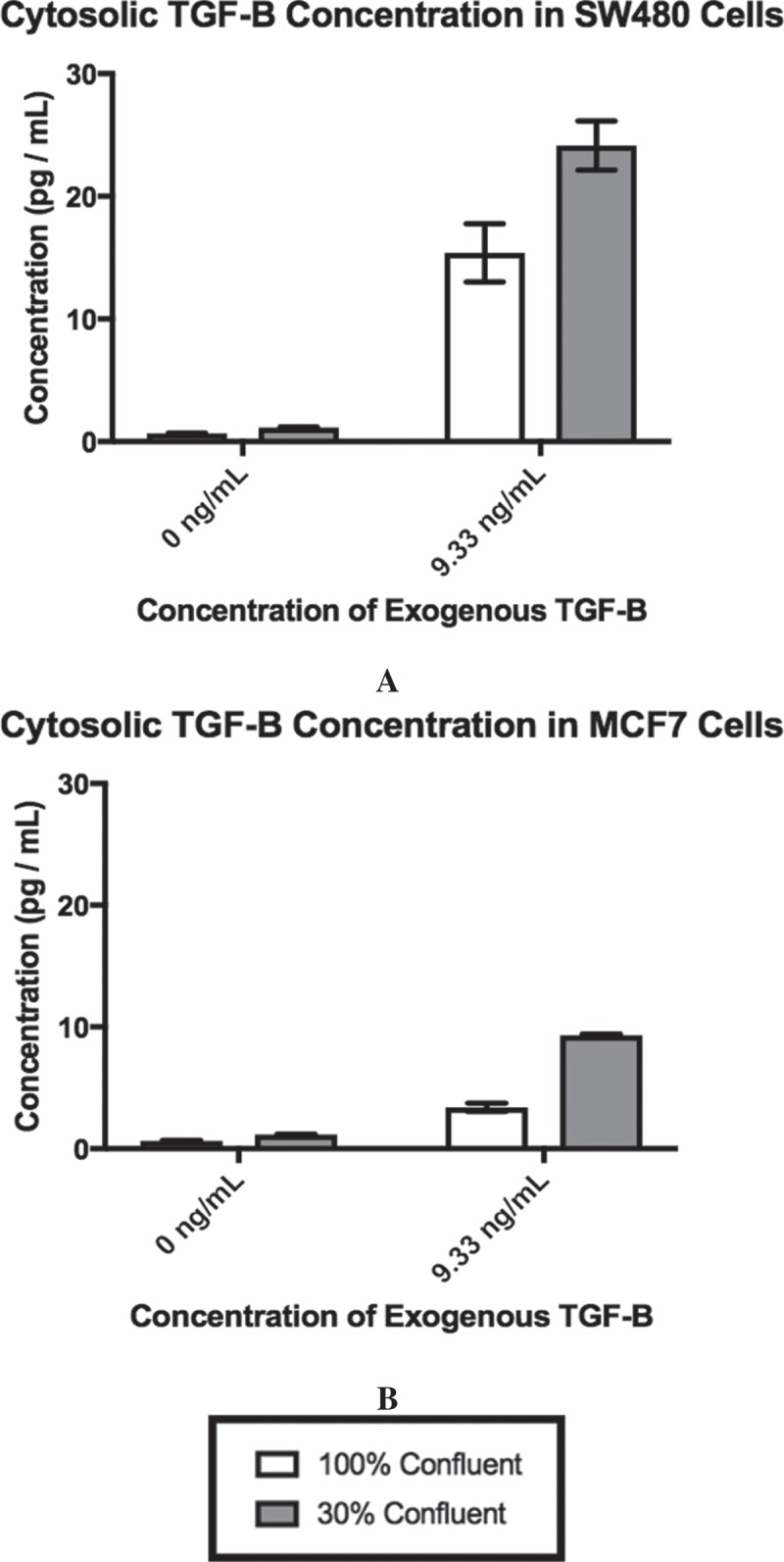
Exogenous TGF-β is required for cellular confluence to impact cytosolic TGF-β. Concentrations for cytosolic TGF-β for each cell line are derived from 1 × 10^6^ cells by ELISA. As exogenous TGF-β is added and confluence is reduced, the cytosolic concentration of TGF-β rises, until it saturates at a concentration of ~25 pg/mL for SW480 cells (A) and ~9 pg/mL for MCF7 cells (B). Using α = 0.05, it was found that the individual effects of confluence (*p < 0.0001*) and exogenous TGF-β (*p < 0.0001*), as well as the interaction between the two factors (SW480, *p = 0.0001*; MCF7, *p < 0.0001*), significantly influenced the concentration of cytosolic TGF-β in both SW480 and MCF7 cell lines. Mean ± standard error of the mean (SEM) is represented.

We hypothesized that cell-cell contact counteracts the activation of EMT by exogenous TGF-β. To test this hypothesis, we used an ELISA to measure the level of cytoplasmic TFG-β generated by SW480 and MCF7 cells in response to exogenous TGF-β when the cells were at different levels of confluence. Epithelial cells can internalize TGF-β molecules following binding to TGF-β cellular receptors by endocytosing the receptors through clathrin-coated vesicles [33]. Additionally, epithelial cells are capable of producing their own TGF-β [33,34]. Therefore, any measured intracellular TGF-β is a combination of internalized exogenous TGF-β and TGF-β synthesized by the cell. The concentration of TGF-β was derived from 1 × 10^6^ cells for both SW480 (Figure 1(a)) and MCF7 (Figure 1(b)) cells. Prior to the addition of exogenous TGF-β, both cell lines have a low level of cytoplasmic TGF-β1 present. The addition of exogenous TGF-β and changes in confluence significantly increased the concentration of TGF-β (SW480, *p = 0.0001*; MCF7, *p < 0.0001*). Interestingly, while their behavior is similar, the SW480 cells have 2-3x as much cytosolic TGF-β present as the MCF7 cells in the 30% confluent +9.33 ng/mL exogenous TGF-β treatment group.

Using a cellular volume of 1.98 × 10^−9^mL [35], we calculated that the average number of TGF-β molecules in a cell. For SW480 cells, in the 100% confluent +0 ng/mL TGF-β culture there are 3.13 × 10^−8^ molecules per cell of cytosolic TGF-β. As confluence is reduced and exogenous TGF-β is applied, this cytosolic TGF-β value increases to 1.15 × 10^−6^ molecules per cell in the 30% confluent +9.33 ng/mL TGF-β. Likewise, for the MCF7 cells, a cell in the 100% confluent +0 ng/mL TGF-β treatment group begins with 2.99 × 10^−8^ molecules per cell, which is then increased to 4.5 × 10^−7^ molecules per cell in the 30% confluent +9.33 ng/mL TGF-β treatment group. The data indicate that the reduction in confluence of the cells and the addition of exogenous TGF-β can work together to impact the cellular response. However, while there is an increase in the overall concentration of TGF-β present, it is still less than one molecule of TGF-β per cell, suggesting that it is a subpopulation of cells experiencing an increase in cytosolic TGF-β.

### Downstream TGF-β pathway targets depend on cellular confluence and exogenous TGF-β

**Figure 2.**
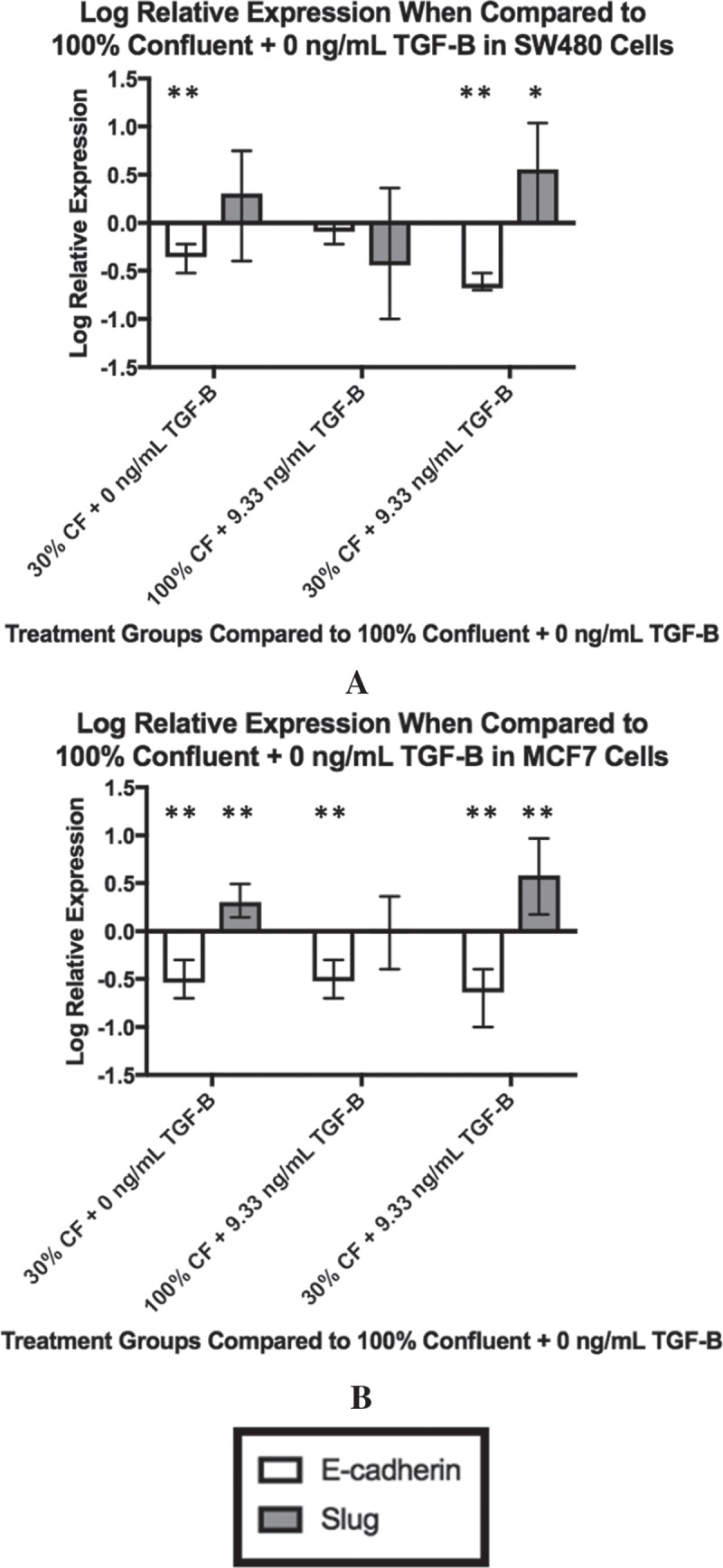
Changing confluence and TGF-β alters downstream gene expression. The expression of downstream targets of TGF-β, E-cadherin and Slug, were measured by qPCR. For both cell lines, the log relative expression is shown of cells under different conditions when compared to the 100% confluent +0 ng/mL TGF-β using α = 0.05. In both the SW480 cells (a) and the MCF7 cells (b), when both confluence is reduced and TGF-β is added, we observe a significant reduction in E-cadherin expression (*p < 0.0001* for the SW480 cells, *p = 0.01* for the MCF7 cells) and a significant increase in Slug expression (*p = 0.016* for the SW480 cells, *p = 0.002* for the MCF7 cells). These changes are also shown in cells with both a reduction in confluence and the addition of exogenous TGF-β (30% confluent + 9.33 ng/mL TGF-β). For all plots, ** indicates *p < 0.01* significance while * indicates *p < 0.05* significance. Data presented is Mean + Standard Error Range.

With the changes in intracellular TGF-β levels based on cell-cell contact and exogenous TGF-β reported by the ELISAs, we investigated if those variations translated into altered expressions of downstream targets in the TGF-β pathway. Analysis using qPCR was used to measure the change in relative expression of Slug, a transcription factor that is a downstream target of TGF-β. Further, as E-cadherin is a classic marker of epithelial cells and can be inhibited with an increase in Slug activity, we measured the relative change in E-cadherin as well. Figure 2(a) shows the log relative expression of different SW480 treatment groups to the 100% confluent +0 ng/mL exogenous TGF-β cells, which would be considered the most epithelial baseline. When only confluence is changed, there is only a statistically significant decrease in E-cadherin expression (*p* < 0.0001), as shown by the 30% confluent +0 ng/mL TGF-β treatment group. It is not until both confluence is reduced and exogenous TGF-β is added that we observe both a statistically significant decrease in E-cadherin (*p < 0.0001*) and an increase in slug (*p = 0.016)*, as shown in the 30% confluent +9.33 ng/mL TGF-β treatment group).

Similar analysis via qPCR was performed on the MCF7 cells and is shown in Figure 2(b). While a reduction in confluence alone was not enough to see an increase in the relative expression of Slug in SW480 cells, this is untrue for the MCF7 cells. As shown in Figure 2(b), when compared to the 100% confluent +0 ng/mL TGF-β cells, the 30% confluent + 0 ng/mL TGF-β cells have an increased relative expression of Slug (*p = 0.006*) and a decreased relative expression on E-cadherin (*p = 0.002*). Once TGF-β is added and confluence is reduced simultaneously, like the SW480 cells, the MCF7 cells undergo a statistically significant decrease in E-cadherin (*p* = 0.01) and a significantly significant increase in Slug (*p *= 0.002). These results indicate that, while the downstream targets of the two cell lines reacted differently to the individual factors, the collaborative influence of exogenous TGF-β and cellular confluence resulted in significant changes in Slug and E-cadherin expression in both cell lines.

### Lack of cell-to-cell contact helps cells exhibit mesenchymal phenotype with application of TGF-β

**Figure 3.**
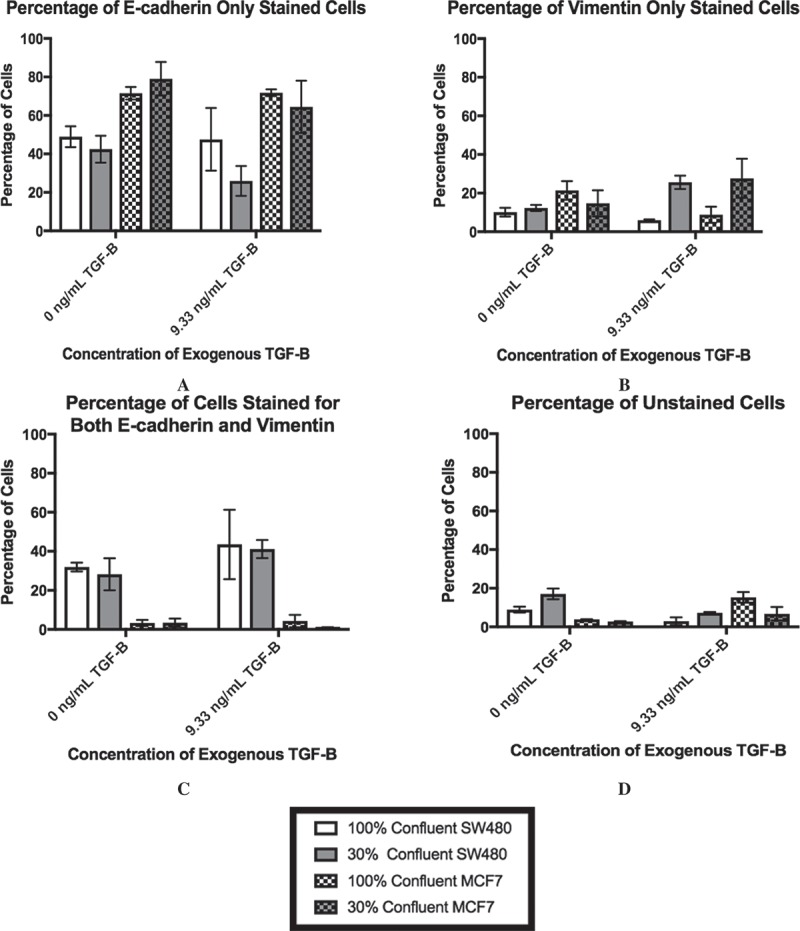
Lower cell-cell contact results in increased mesenchymal cell markers with the addition of TGF-β. SW480 and MCF7 cells were analyzed for epithelial (E-cadherin) or mesenchymal (vimentin) markers by flow cytometry. Cells were gated for staining for only E-cadherin (a), only vimentin (b), both markers (c), and neither marker (d) during flow cytometry experiments. Mean ± standard error of the mean (SEM) is represented. Without the application of exogenous TGF-β, changes in confluence lead to relatively small (although statistically significant) changes in the protein expression in both cell lines. With extracellular TGF-β, changes in confluence strongly affect the stained subpopulations (p < 0.0001). Despite this similarity, the cell staining patterns are very different between cell lines. The SW480 cells display a group of cells that stain for both E-cadherin and vimentin. Meanwhile, both before and after the reduction in confluence and the addition of TGF-β, the MCF7 cell population stains primarily for E-cadherin only.

As the ELISA data implied that a subpopulation of cells may be driving the changes in TGF-β levels, we analyzed the cultures via flow cytometry to measure cellular markers on an individual cell basis. The results of this staining are shown in Figure 3. We chose E-cadherin and vimentin as our markers for the epithelial and mesenchymal phenotypes, respectively. E-cadherin is a classical marker of the epithelial phenotype due to its involvement in cell-to-cell adhesion while vimentin has been associated with the mesenchymal phenotype and invasive behavior [36,37]. Therefore, an E-cadherin-only stained cell (Figure 3(a)) would be indicative of the epithelial phenotype while vimentin-only staining (Figure 3(b)) would suggest that the cell had adopted the mesenchymal phenotype. At 100% confluence +0 ng/mL, the MCF7 population is mostly comprised of cells that stained for E-cadherin-only, while the SW480 cells show diverse staining patterns that is divided up between all four staining subgroups. In particular, there is a subpopulation of the SW480 cells that stained for both E-cadherin and vimentin, indicating that the cells have both epithelial and mesenchymal qualities (Figure 3(c)). Thus, while the subpopulation break-down of the two cell lines differs greatly, without the application of exogenous TGF-β, changes in confluence lead to relatively small (although statistically significant) changes in the protein expression in both cell lines. On the other hand, with extracellular TGF-β, changes in confluence strongly affect the stained subpopulations (p < 0.0001). Specifically, we observe a decrease in the E-cadherin only population (Figure 3(a)) and an increase in the vimentin only population of the SW480 cells (Figure 3(b)). The subpopulation of cells that expressed both markers, however, remained unchanged. Conversely, while the MCF7 cells also show a rise in the subpopulation that expressed vimentin-only staining (Figure 3(b)), the majority of the cell population still stained positively for E-cadherin only (Figure 3(a)), indicating that the MCF7 cells at 30% confluent +9.33 ng/mL TGF-β were still highly epithelial.

**Figure 4.**
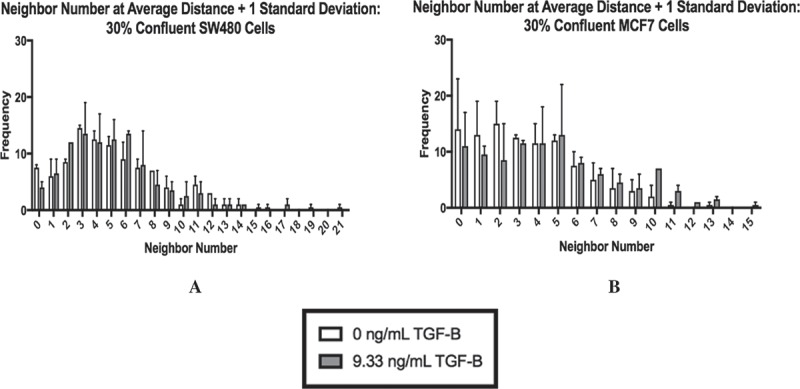
The neighbor number for 30% confluent cells at a threshold of the average nuclei distance +1 standard deviation are shown for SW480 (a) and MCF7 (b) cells, respectively. At this distance, both cell lines skew right with cells having few neighbors. Mean ± standard error of the mean (SEM) is represented.

To further investigate the influence of cellular contact on the subpopulations, we used immunocytochemistry staining to visualize E-cadherin and vimentin proteins. To quantify the cell-cell contact, we needed to develop criteria to determine the number of neighbors a cell had in a given field of view. First, we established the nuclei threshold distance, and 100 cells were then randomly selected from each treatment group (and technical replicates) using MATLAB. Due to the large standard deviation, this process was carried out at a threshold of the average nuclei distance +1 standard deviation (shown in Figure 4), as well as the average nuclei distance, and the average nuclei distance +2 standard deviations (see Supplementary Figure 3). As shown in Figure 4, at 30% confluence, using the threshold of the average distance + 1 standard deviation, the populations of cells skew right, indicating that the majority of cells had only a few neighbors at both 0 ng/mL TGF-β and 9.33 ng/mL TGF-β. In fact, the majority of the SW480 cells at both concentrations of TGF-β had 3–5 neighbors (Figure 4(a)) while the majority of MCF7 cells had 0–5 neighbors (Figure 4(b)).

Once the range of possible neighbors for cells was determined, we sought to understand if the number of neighbors a cell had was related to its likelihood to undergo EMT. Thus, the cellular neighbor number range for the 30% confluent cells was divided into 3 categories for each cell type: low neighbor number (0–2 neighbors), medium neighbor number (3–5 neighbors), and high neighbor number (6+ neighbors). For each category, 25 cells (or the maximum number of cells in that category if it was less than 25) were randomly selected and categorized based on their staining: E-cadherin-only, vimentin-only, dual staining, and neither. Similar to the flow cytometry staining, these markers tell us if a cell can be categorized as an epithelial cell, a mesenchymal cell, a cell existing in an intermediary state, or simply did not stain.

**Figure 5.**
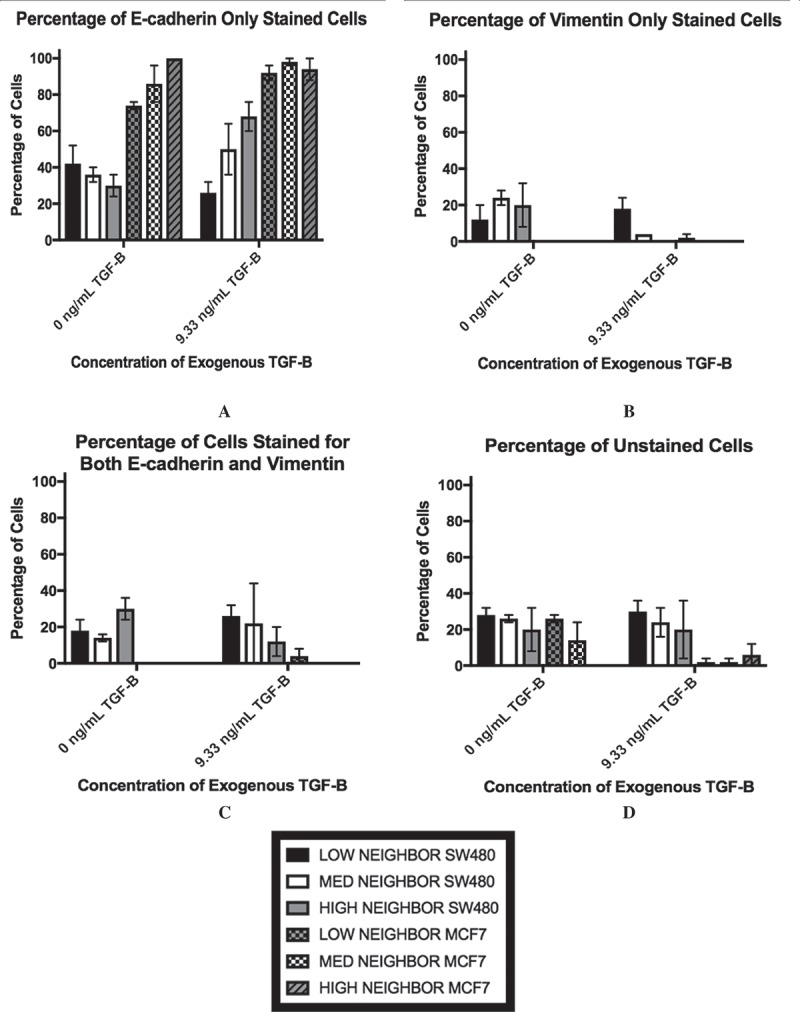
The percentage of SW480 cells that stained for E-cadherin only (a), vimentin only (b), both markers (c), and neither marker (d) in cell populations that are 30% confluent during immunocytochemistry are shown. The distance threshold is the average neighbor distance +1 standard deviation for each cell line is shown. Like the flow cytometry results, the existence of a dual-staining population of SW480 cells is apparent while the MCF7 cells lack this subpopulation. Further, at all three levels of cellular contact, both with and without exogenous TGF-β, the MCF7 cells exhibit a high amount of staining for E-cadherin only.

**Figure 6.**
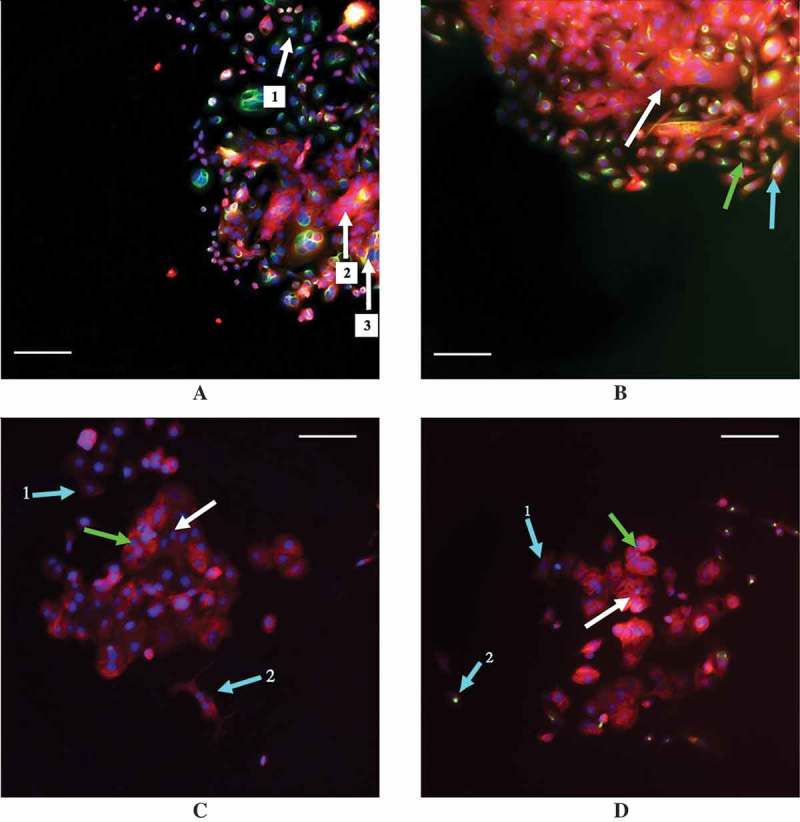
Examples of staining in the MCF7 and SW480 cell lines with E-cadherin staining in red and vimentin staining in green. (a) 30% confluent SW40 cells with 0 ng/mL TGF-β while (b) is 30% confluent SW480 cells with 9.33 ng/mL TGF-β. (c) 30% confluent MCF7 cells with 0 ng/mL TGF-β while (d) is 30% confluent MCF7 cells with 9.33 ng/mL TGF-β. Arrows are color coordinated: blue arrows indicate a cell with a low number of neighbors, green indicates cells with a medium number of neighbors, and white indicates cells with a high number of neighbors, as determined using a threshold distance of the average nuclei distance +1 standard deviation. A scale bar of 100 μm is shown in each image. (a) Arrows 1, 2, and 3 all indicate three different SW480 cells that were classified as having a high number of neighbors that all stained differently: cell 1 has stained solely for vimentin, cell 2 for E-cadherin only, and cell 3 has stained for both vimentin and E-cadherin simultaneously. (b) The SW480 30% confluent +9.33 ng/mL TGF-β treatment group and cells that are classified as having different neighbor numbers all staining dually for E-cadherin and vimentin. (c) The cell at arrow 1 has a low neighbor number and has stained faintly for E-cadherin while the cell at arrow 2 also has a low neighbor number and has stained slightly more for E-cadherin, despite the fact that it is beginning to adopt a spindle-like phenotype. Additionally, E-cadherin is also expressed in the cells at the green arrow and the white arrow, which have a medium number of neighbors and a high number of neighbors, respectively. (d) With the addition of 9.33 ng/mL exogenous TGF-β, MCF7 cells with a medium (green arrow) and high (white arrow) neighbor number are positively stained for E-cadherin. Arrow 1 indicates a cell with a low neighbor number that has not stained for either E-cadherin or vimentin, or possibly faintly stained for E-cadherin, while arrow 2 points to a cell that also has a low neighbor number that has positively stained for vimentin.

The staining analysis for different neighbor number categories of the 30% confluent SW480 cells and the 30% confluent MCF7 cells using a distance threshold of the average nuclei distance +1 standard deviation are shown in Figure 5. The SW480 cells exhibit variability in their staining, as shown by the staining for all four categories in all treatment groups. Examples of this variability in SW480 staining are shown in Figure 6(a,b), with E-cadherin positive staining in red and vimentin positive staining in green. However, with the addition of TGF-β, the SW480 cells with a few number of neighbors are those most likely to exhibit vimentin-only staining (Figure 5(b)). The MCF7 cells, on the other hand, exhibit far less variability. At least 70% of the MCF7 cells in each neighbor number category stained positively for E-cadherin only, whether or not TGF-β was present (Figure 5(a)). Examples of MCF7 staining are shown in Figure 6(c,d) (again, E-cadherin positive staining red, vimentin positive staining green). Despite this overwhelming E-cadherin only staining, the cells with low cellular contact are those most likely to exhibit either vimentin only staining or dual E-cadherin and vimentin staining upon the addition of exogenous TGF-β (Figure 5(b,c)).

Note that the overall fluorescence expression between the results of the flow cytometry experiments and the immunocytochemistry experiments do differ, a result that could be due to differences in sensitivity to the techniques or the growing patterns of the cells, as they were prone to grow on top of each other. However, despite these differences, together, the results show that with the addition of 9.33 ng/mL TGF-β, cells with limited cell-to-cell contact are those most likely to be exhibiting mesenchymal cell-associated staining in both cell lines.

## Discussion

With the binding of exogenous TGF-β to surface receptors, epithelial cells are able to endocytose TGF-β while also producing their own TGF-β to create a positive feedback loop [33,34]. Therefore, measuring the concentration of cytosolic TGF-β helps assess the level of TGF-β activation within the cells. Using the volume of a single epithelial cell and the concentrations of TGF-β, we estimated that there was <1 TGF-β molecule per cell in the entire population. This finding would suggest that many of the cells in each treatment group either: (1) did not have the TGF-β bind to its cellular receptors, (2) did not endocytose the TGF-β that did bind to its receptors, or (3) endocytosed the exogenous TGF-β which was then degraded and no additional TGF-β was produced by the cell, all of which would mean that the TGF-β pathway was not activated in these cells. Thus, any changes that occurred in the concentration of cytosolic TGF-β were driven by a small subset of cells in the population.

As the confluence was reduced at 0 ng/mL TGF-β, both cell lines saw a slight increase in the concentration of cytosolic TGF-β. To understand whether this increase in cytosolic TGF-β corresponded with the activation of the TGF-β pathway, qPCR analysis was used to measure the changes in relative expression of Slug and E-cadherin, the transcription factor and protein that are downstream targets of TGF-β, respectively. For the SW480 cells, this change in cytosolic concentration was not associated with significant changes in the expression of Slug mRNA or E-cadherin mRNA. For the MCF7 cells, however, the increase in cytosolic TGF-β was associated with a significant increase in Slug mRNA expression and a significant decrease in E-cadherin mRNA expression, indicating that intracellular EMT associated pathways had been activated. However, as shown by the flow cytometry, even as confluence was decreased, at 0 ng/mL TGF-β, the MCF7 cell population was mostly still E-cadherin-only positive in their staining, indicating that the population was still highly epithelial in nature.

When exogenous TGF-β was added and confluence was reduced simultaneously, both the SW480 population and the MCF7 population saw a large increase in the cytosolic concentration of TGF-β present. For both cell lines, this increase in cytosolic TGF-β was associated with a significant increase in Slug mRNA expression and a significant decrease in E-cadherin mRNA expression, indicating that the TGF-β pathway had been activated. Additionally, with the exogenous TGF-β, changes in confluence strongly affected the staining of the subpopulations for both cell lines, as shown in the flow cytometry analysis. In the case of the SW480 cells, due to the unchanging subpopulation of dual E-cadherin and vimentin staining cells, it can be inferred that as cells move away from the epithelial phenotype, they pass through an intermediate stage before adopting mesenchymal characteristics. For the MCF7 cells, the lack of an intermediate subpopulation suggests that the subpopulation causing significant changes in staining are the cells that are moving directly from the epithelial to the mesenchymal phenotype. Moreover, immunocytochemistry staining shows that the MCF7 cells most likely to cause these changes are those with little cell-to-cell contact, suggesting that a higher degree of cell-to-cell contact inhibits the move towards the mesenchymal phenotype for this cell line.

This work offers insight into the upregulation of EMT in two different cell lines. Primarily, the differences between the two cell lines studied highlight that there is not a universal process by which EMT is upregulated. Heterogeneity within a tumor has been previously reported and the level of heterogeneity can differ from tumor to tumor [38], making the option of pursuing a common treatment plan unlikely. One potential extension of this work should include exploring how TGF-β receptor expression and production of TGF-β following activation are influenced by cellular contact. Conducting these studies both within the treatment groups presented in this work, and in less confluent cell cultures, could help show how position within a tumor influences cellular response to EMT activation via TGF-β signaling.

The two cell lines investigated in this work each provide specific insight as to further avenues of exploration. The MCF7 cell data, in particular, suggests that, during low exposure to TGF-β, treatment should focus on cells at the edge of a tumor, as that is where non-epithelial cells are most likely to exist. By preventing these cells from transitioning, it could be possible to keep cells closer to the center of the tumor from transitioning due to their initial epithelial nature and their adhesion with surrounding cells. These findings affirm the predictions of our recent computational model of EMT [12]. Further, the highly epithelial nature of the MCF7 cells, despite the addition of TGF-β, suggests that there was not enough exogenous TGF-β added to the cell cultures to push the cells towards phenotypic and behavioral changes. However, previous studies have shown that MCF7 cells can be induced to transition into mesenchymal-like cells if two different stimuli are applied simultaneously, as in the work carried out by Walsh and Damjanovski [39]. In this study, insulin-like growth factor (IGF) was applied to MCF7 cells prior to the addition of TGF-β, a combination that resulted in EMT [39]. The results of their work in conjunction with the work presented here suggest that an important future experiment would be to investigate if the activation of other EMT-related paths, such as the Wnt pathway, in addition to the TGF-β pathway would result in a larger subpopulation of MCF7 cells undergoing EMT.

Unlike the MCF7 cells, in the SW480 cells we observed a transition phase where both epithelial and mesenchymal proteins are co-expressed before they become fully mesenchymal. This intermediate phase of EMT has been previously reported in other studies and has been found to exist in circulating tumor cells. Described as a ‘metastable’ cellular state, these cells are thought to have the flexibility to promote or reverse the epithelial to mesenchymal transition [38]. These previous studies and the work presented here together suggest reversing the phenotype of this metastable transitional state could be a viable therapeutic strategy before these cells metastasize at a distant site.
