# Supplementary material for: The role of cellular contact and TGF-beta signaling in the activation of the epithelial mesenchymal transition (EMT)
Source: Cell Adh Migr. 2018 Oct 8;13(1):63–75. doi: 10.1080/19336918.2018.1526597 (PMC6527395; doi:10.1080/19336918.2018.1526597)
Supplement: Supplemental Material [file kcam-13-01-1526597-s001.docx]

| **Supplementary Table 1: Two-way ANOVA for Cytosolic TGF-β in SW480 Cells** | | | | | |
| --- | --- | --- | --- | --- | --- |
|  | **SS** | **DF** | **MS** | **F** | **p-value** |
| **Interaction** | 302.0 | 4 | 75.50 | 6.508 | 0.0003 |
| **TGF-β** | 3859 | 2 | 1929 | 166.3 | < 0.0001 |
| **Confluence** | 374 | 2 | 187.0 | 16.12 | < 0.0001 |
| **Residual** | 510.5 | 44 | 11.60 |  |  |
| **Supplementary Table 2: Two-way ANOVA for Cytosolic TGF-β in MCF7 Cells** | | | | | |
|  | **SS** | **DF** | **MS** | **F** | **p-value** |
| **Interaction** | 66.00 | 4 | 16.50 | 12.19 | < 0.0001 |
| **TGF-β** | 331.3 | 2 | 165.70 | 122.4 | < 0.0001 |
| **Confluence** | 59.09 | 2 | 29.54 | 21.82 | < 0.0001 |
| **Residual** | 59.57 | 44 | 1.354 |  |  |

| **Supplementary Table 3: REST Analysis for Gene Expression in SW480 Cells** | | | | | | |
| --- | --- | --- | --- | --- | --- | --- |
| **Control** | **Treatment Group** | **Gene** | **Relative Expression** | **Std. Error Range** | **p-value** | **Expression** |
| 100% CF 0 ng/mL TGF-β | 60% CF 0 ng/mL TGF-β | E-cadherin | 0.55 | (0.4-0.8) | < 0.0001 | Down |
|  |  | Slug | 0.89 | (0.2-2.7) | 0.747 | -- |
| 100% CF 0 ng/mL TGF-β | 30% CF 0 ng/mL TGF-β | E-cadherin | 0.44 | (0.3-0.6) | < 0.0001 | Down |
|  |  | Slug | 2.01 | (0.4-5.6) | 0.140 | -- |
| 100% CF 0 ng/mL TGF-β | 100% CF 3 ng/mL TGF-β | E-cadherin | 0.43 | (0.3-0.7) | < 0.0001 | Down |
|  |  | Slug | 0.31 | (0.1-1.4) | 0.023 | Down |
| 100% CF 0 ng/mL TGF-β | 60% CF 3 ng/mL TGF-β | E-cadherin | 0.41 | (0.2-0.7) | < 0.0001 | Down |
|  |  | Slug | 0.58 | (0.1-1.8) | 0.241 | -- |
| 100% CF 0 ng/mL TGF-β | 30% CF 3 ng/mL TGF-β | E-cadherin | 0.17 | (0.1-0.4) | < 0.0001 | Down |
|  |  | Slug | 2.25 | (0.8-10.1) | 0.116 | -- |
| 100% CF 0 ng/mL TGF-β | 100% CF 9.33 ng/mL TGF-β | E-cadherin | 0.81 | (0.6-1.0) | 0.124 | -- |
|  |  | Slug | 0.36 | (0.1-2.3) | 0.055 | -- |
| 100% CF 0 ng/mL TGF-β | 60% CF 9.33 ng/mL TGF-β | E-cadherin | 0.39 | (0.2-0.8) | < 0.0001 | Down |
|  |  | Slug | 0.76 | (0.2-2.2) | 0.57 | -- |
| 100% CF 0 ng/mL TGF-β | 30% CF 9.33 ng/mL TGF-β | E-cadherin | 0.21 | (0.2-0.3) | < 0.0001 | Down |
|  |  | Slug | 3.6 | (1.0-10.9) | 0.016 | Up |

| **Supplementary Table 4: Further REST Analysis for Gene Expression in SW480 Cells** | | | | | | |
| --- | --- | --- | --- | --- | --- | --- |
| **Control** | **Treatment Group** | **Gene** | **Relative Expression** | **Std. Error Range** | **p-value** | **Expression** |
| 60% CF 0 ng/mL TGF-β | 60% CF 3 ng/mL TGF-β | E-cadherin | 0.75 | (0.5-1.3) | 0.122 | -- |
|  |  | Slug | 0.65 | (0.5-1.0) | 0.008 | Down |
| 60% CF 0 ng/mL TGF-β | 60% CF 9.33 ng/mL TGF-β | E-cadherin | 0.72 | (0.4-1.4) | 0.129 | -- |
|  |  | Slug | 0.85 | (0.5-1.5) | 0.404 | -- |
| 30% CF 0 ng/mL TGF-β | 30% CF 3 ng/mL TGF-β | E-cadherin | 0.39 | (0.2-0.9) | 0.002 | Down |
|  |  | Slug | 1.12 | (0.4-3.1) | 0.809 | -- |
| 30% CF 0 ng/mL TGF-β | 30% CF 9.33 ng/mL TGF-β | E-cadherin | 0.49 | (0.4-0.6) | 0.001 | Down |
|  |  | Slug | 1.79 | (1.0-3.3) | 0.036 | Up |
| 100% CF 3 ng/mL TGF-β | 60% CF 3 ng/mL TGF-β | E-cadherin | 0.95 | (0.5-1.9) | 0.704 | -- |
|  |  | Slug | 1.87 | (0.6-5.5) | 0.189 | -- |
| 100% CF 3 ng/mL TGF-β | 30% CF 3 ng/mL TGF-β | E-cadherin | 1.83 | (0.6-5.9) | 0.159 | -- |
|  |  | Slug | 7.27 | (1.2-39.3) | 0.116 | -- |
| 100% CF 9.33 ng/mL TGF-β | 60% CF 9.33 ng/mL TGF-β | E-cadherin | 0.49 | (0.3-0.8) | 0.001 | Down |
|  |  | Slug | 2.09 | (0.5-10.1) | 0.201 | -- |
| 100% CF 9.33 ng/mL TGF-β | 30% CF 9.33 ng/mL TGF-β | E-cadherin | 0.26 | (0.2-0.3) | < 0.0001 | Down |
|  |  | Slug | 9.90 | (2.0-51.4) | 0.001 | Up |

| **Supplementary Table 5: REST Analysis for Gene Expression in MCF7 Cells** | | | | | | |
| --- | --- | --- | --- | --- | --- | --- |
| **Control** | **Treatment Group** | **Gene** | **Relative Expression** | **Std. Error Range** | **p-value** | **Expression** |
| 100% CF 0 ng/mL TGF-β | 60% CF 0 ng/mL TGF-β | E-cadherin | 0.14 | (0.1-0.3) | < 0.0001 | Down |
|  |  | Slug | 1.67 | (1.3-2.1) | 0.001 | Up |
| 100% CF 0 ng/mL TGF-β | 30% CF 0 ng/mL TGF-β | E-cadherin | 0.29 | (0.2-0.5) | 0.002 | Down |
|  |  | Slug | 2.01 | (1.4-3.1) | 0.006 | Up |
| 100% CF 0 ng/mL TGF-β | 100% CF 3 ng/mL TGF-β | E-cadherin | 0.54 | (0.3-0.9) | 0.001 | Down |
|  |  | Slug | 1.37 | (0.6-3.1) | 0.333 | -- |
| 100% CF 0 ng/mL TGF-β | 60% CF 3 ng/mL TGF-β | E-cadherin | 0.31 | (0.2-0.5) | < 0.0001 | Down |
|  |  | Slug | 3.37 | (2.7-4.3) | 0.002 | Up |
| 100% CF 0 ng/mL TGF-β | 30% CF 3 ng/mL TGF-β | E-cadherin | 0.21 | (0.1-0.4) | < 0.0001 | Down |
|  |  | Slug | 3.76 | (2.8-4.8) | 0.001 | Up |
| 100% CF 0 ng/mL TGF-β | 100% CF 9.33 ng/mL TGF-β | E-cadherin | 0.30 | (0.2-0.5) | 0.004 | Down |
|  |  | Slug | 1.01 | 0.4-2.3) | 0.963 | -- |
| 100% CF 0 ng/mL TGF-β | 60% CF 9.33 ng/mL TGF-β | E-cadherin | 0.27 | (0.2-0.4) | 0.001 | Down |
|  |  | Slug | 2.45 | (2.0-3.0) | 0.002 | Up |
| 100% CF 0 ng/mL TGF-β | 30% CF 9.33 ng/mL TGF-β | E-cadherin | 0.23 | (0.1-0.4) | 0.01 | Down |
|  |  | Slug | 3.81 | (1.5-9.3) | 0.002 | Up |

| **Supplementary Table 6: Further REST Analysis for Gene Expression in MCF7 Cells** | | | | | | |
| --- | --- | --- | --- | --- | --- | --- |
| **Control** | **Treatment Group** | **Gene** | **Relative Expression** | **Std. Error Range** | **p-value** | **Expression** |
| 60% CF 0 ng/mL TGF-β | 60% CF 3 ng/mL TGF-β | E-cadherin | 2.24 | (1.0-4.8) | 0.014 | Up |
|  |  | Slug | 2.03 | (1.7-2.5) | 0.001 | Up |
| 60% CF 0 ng/mL TGF-β | 60% CF 9.33 ng/mL TGF-β | E-cadherin | 1.93 | (1.0-3.6) | 0.005 | Up |
|  |  | Slug | 1.47 | (1.2-1.7) | 0.001 | Up |
| 30% CF 0 ng/mL TGF-β | 30% CF 3 ng/mL TGF-β | E-cadherin | 0.74 | (0.5-1.1) | 0.103 | -- |
|  |  | Slug | 1.87 | (1.2-2.9) | 0.001 | Up |
| 30% CF 0 ng/mL TGF-β | 30% CF 9.33 ng/mL TGF-β | E-cadherin | 0.80 | (0.5-1.2) | 0.206 | -- |
|  |  | Slug | 1.90 | (0.6-4.8) | 0.107 | -- |
| 100% CF 3 ng/mL TGF-β | 60% CF 3 ng/mL TGF-β | E-cadherin | 0.57 | (0.4-0.8) | < 0.0001 | Down |
|  |  | Slug | 2.46 | (1.1-5.6) | 0.007 | Up |
| 100% CF 3 ng/mL TGF-β | 30% CF 3 ng/mL TGF-β | E-cadherin | 0.39 | (0.3-0.6) | < 0.0001 | Down |
|  |  | Slug | 2.74 | (1.2-6.1) | < 0.0001 | Up |
| 100% CF 9.33 ng/mL TGF-β | 60% CF 9.33 ng/mL TGF-β | E-cadherin | 0.87 | (0.8-1.0) | 0.037 | Down |
|  |  | Slug | 2.42 | (1.1-5.4) | 0.008 | Up |
| 100% CF 9.33 ng/mL TGF-β | 30% CF 9.33 ng/mL TGF-β | E-cadherin | 0.75 | (0.6-1.0) | 0.041 | Down |
|  |  | Slug | 3.76 | (0.9-16.1) | 0.041 | Up |

**Flow Cytometry**

| **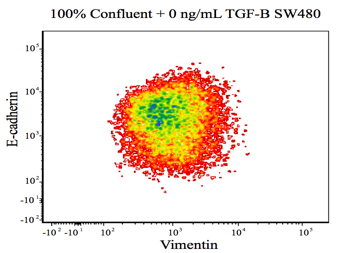** | **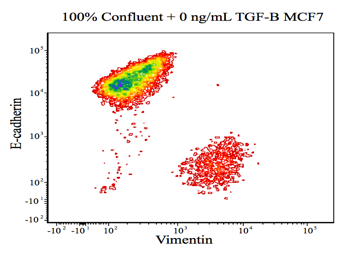** |
| --- | --- |
| **Figure A** | **Figure E** |
| **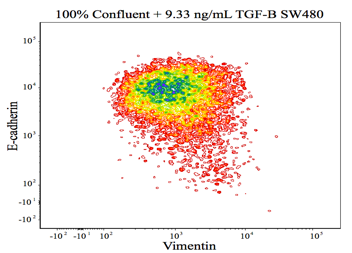** | **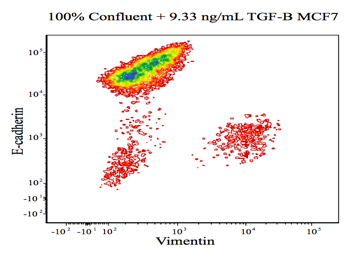** |
| **Figure B** | **Figure F** |
| **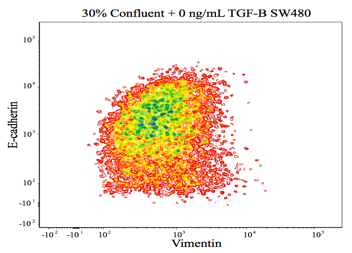** | **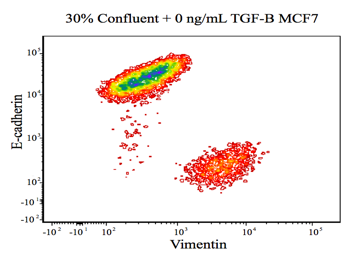** |
| **Figure C** | **Figure G** |
| **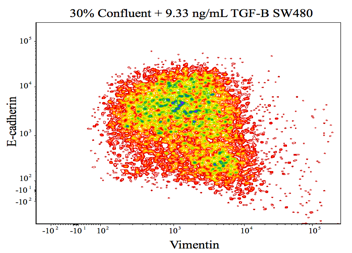** | **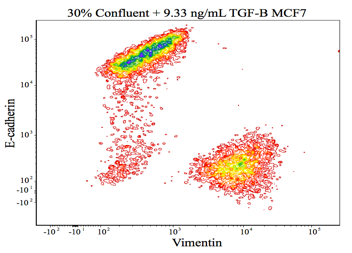** |
| **Figure D** | **Figure H** |
| **Supplementary Figure 1:** The fluorescence intensity of E-cadherin and vimentin on 4 treatment groups are shown, with SW480 cells in A-D and MCF7 cells in E-H. For 100% confluent, the SW480 population did have high E-cadherin fluorescent intensity, as indicated by the dark blue group of cells in the upper left hand quadrant in A and B. As confluence is reduced and TGF*-*β is added, there is a population of cells in the lower right quadrant, indicating an increase in cells that stain vimentin-only, and thus exhibit the mesenchymal phenotype. In E-H, MCF7 cells stained mostly for E-cadherin-only. But, as confluence is reduced and TGF-β is added, the portion of the population that stain vimentin-only grew. | |

**Immunocytochemistry**

| 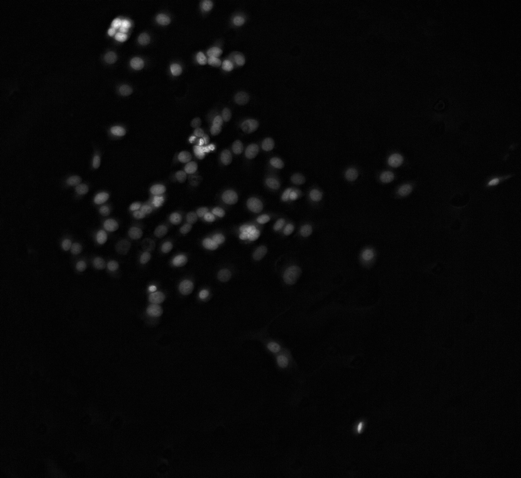 | 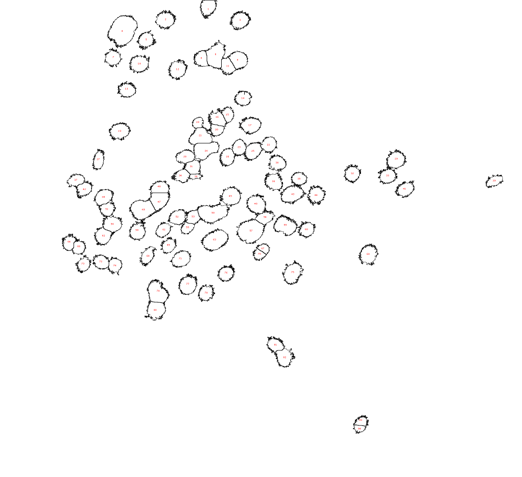 |
| --- | --- |
| **Figure A** | **Figure B** |
| **Supplementary Figure 2:** (A) MCF7 nuclei stained with DAPI where background noise was subtracted using ImageJ. (B) final post processed image of (A) where each cell was given a corresponding numerical identity using ImageJ. The number of neighbors was determined for each cell line using different thresholding nuclei distances. | |

| 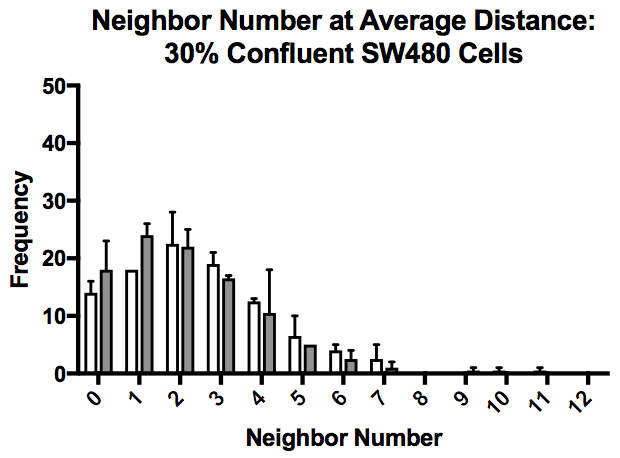 | 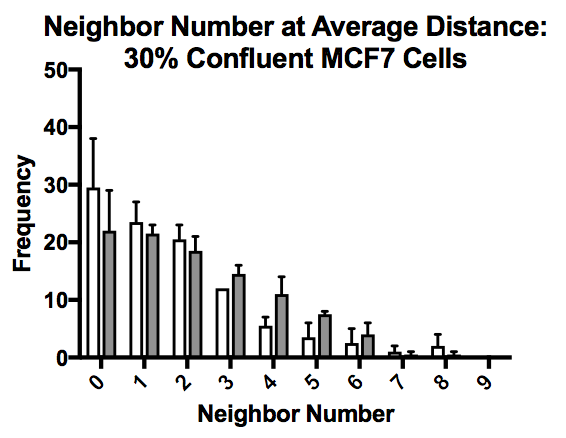 |
| --- | --- |
| **Figure A** | **Figure B** |
| 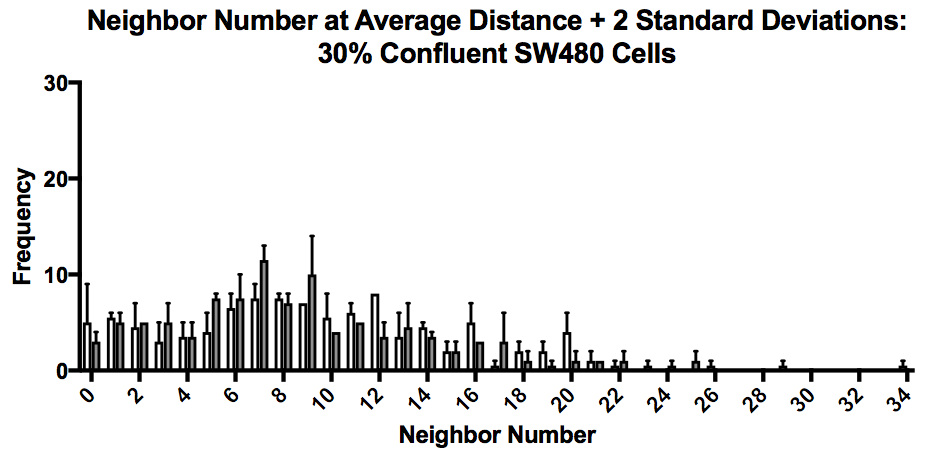 | 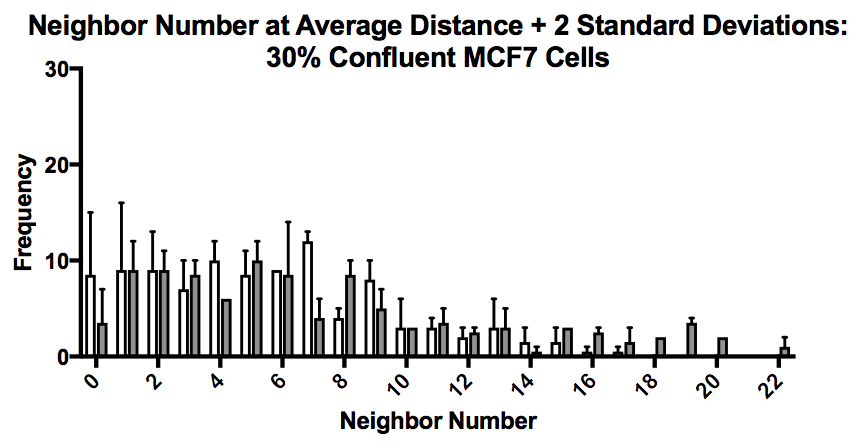 |
| **Figure C** | **Figure D** |
| **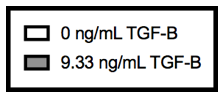** | |
| **Supplementary Figure 3:** The neighbor number for 30% confluent cells at a threshold of the average nuclei distance are shown in (A) and (B) for the SW480 and MCF7 cells, respectively, while the neighbor number for the 30% confluent cells at a threshold of the average nuclei distance + 2 standard deviations are shown in (C) and (D). At both distances, the number of neighbors each cell population has skews right. With an increase in the distance between nuclei to count neighbors, while both cell lines still skew right towards a low neighbor number, each cell line is capable of having a higher number of neighbors, albeit with a lower frequency. Mean + standard error of the mean (SEM) is represented. | |
